# Remarkable stability in patterns of blood-stage gene expression during episodes of non-lethal *Plasmodium yoelii* malaria

**DOI:** 10.1186/1475-2875-11-265

**Published:** 2012-08-06

**Authors:** Amy Cernetich-Ott, Thomas M Daly, Akhil B Vaidya, Lawrence W Bergman, James M Burns

**Affiliations:** 1 Centre for Molecular Parasitology, Department of Microbiology and Immunology, Drexel University College of Medicine, 2900 Queen Lane, Philadelphia, PA, 19129, USA

**Keywords:** Malaria, *Plasmodium yoelii*, Blood-stage parasites, DNA microarrays, In vivo gene expression profiles

## Abstract

**Background:**

Microarray studies using in vitro cultures of synchronized, blood-stage *Plasmodium falciparum* malaria parasites have revealed a ‘just-in-time’ cascade of gene expression with some indication that these transcriptional patterns remain stable even in the presence of external stressors. However, direct analysis of transcription in *P. falciparum* blood-stage parasites obtained from the blood of infected patients suggests that parasite gene expression may be modulated by factors present in the in vivo environment of the host. The aim of this study was to examine changes in gene expression of the rodent malaria parasite, *Plasmodium yoelii* 17X, while varying the in vivo setting of replication.

**Methods:**

Using *P. yoelii* 17X parasites replicating in vivo, differential gene expression in parasites isolated from individual mice, from independent infections, during ascending, peak and descending parasitaemia and in the presence and absence of host antibody responses was examined using *P. yoelii* DNA microarrays. A genome-wide analysis to identify coordinated changes in groups of genes associated with specific biological pathways was a primary focus, although an analysis of the expression patterns of two multi-gene families in *P. yoelii*, the *yir* and *pyst-a* families, was also completed.

**Results:**

Across experimental conditions, transcription was surprisingly stable with little evidence for distinct transcriptional states or for consistent changes in specific pathways. Differential gene expression was greatest when comparing differences due to parasite load and/or host cell availability. However, the number of differentially expressed genes was generally low. Of genes that were differentially expressed, many involved biologically diverse pathways. There was little to no differential expression of members of the *yir* and *pyst-a* multigene families that encode polymorphic proteins associated with the membrane of infected erythrocytes. However, a relatively large number of these genes were expressed during blood-stage infection regardless of experimental condition.

**Conclusions:**

Taken together, these results indicate that 1) *P. yoelii* gene expression remains stable in the presence of a changing host environment, and 2) concurrent expression of a large number of the polymorphic *yir* and *pyst-a* genes, rather than differential expression in response to specific host factors, may in itself limit the effectiveness of host immune responses.

## Background

Of the four species of *Plasmodium* that infect humans, *Plasmodium falciparum* is most commonly associated with severe disease and mortality, especially in young children. Immunity to this species does develop, but requires repeated exposure over many years [1]. Sequencing and annotation of the *P. falciparum* genome revealed ~5,300 genes, many of which are predicted to encode proteins involved in host cell invasion and immune evasion, but the function of a large proportion remains unknown [2,3]. A detailed microarray study of gene expression using blood-stage *P. falciparum* parasites cultured in vitro showed that a large percentage of the genome is expressed during the asexual stage, and that there is a surprising ‘continuous cascade’ of gene expression. Most genes are expressed only once during the asexual cycle and groups of genes involved in similar processes are active at the same time [4,5]. This highly coordinated expression profile implies tightly controlled regulation, although as relatively few transcription factors have been identified in *Plasmodium*, precisely how gene regulation occurs remains incompletely understood. In these transcriptional studies, in vitro cultured *P. falciparum* parasites were tightly synchronized to evaluate gene expression at distinct points during the life cycle, and growth conditions were controlled to avoid introducing unwanted stressors and other confounding factors.

Transcriptional evaluation using malaria parasites isolated from infected individuals is more technically challenging and is influenced by the diversity of both host and parasite, their interaction and the ensuing immune response. Additional environmental factors such as body temperature, nutritional status and hormone levels [6] also vary considerably between individuals and may influence parasite growth, gene expression and ultimately disease outcome. In one study, a transcriptional analysis of ring-stage parasites isolated directly from human subjects revealed distinct transcriptional profiles, thought by the authors to occur in response to the in vivo environment and not necessarily detectible using parasites cultured in vitro [7]. There has been some debate as to the reasons for the observed differences [8]. More such studies are necessary to fully examine parasite gene expression patterns in vivo and to determine how they change in response to host factors and immune pressures.

The genomes of *Plasmodium* parasites contain a number of multi-gene families, including the relatively well-studied *var* genes of *P. falciparum* which encode ~60 antigenic variants of *P. falciparum* erythrocyte membrane protein-1 (*Pf* EMP-1). *Pf* EMP-1 is expressed on the surface of the infected red blood cell (iRBC) membrane, and mediates binding to a variety of host endothelial cell receptors in tissues such as brain, placenta, lung and kidney [9]. Interestingly, the *var* genes appear to be unique to *P* . *falciparum* and possibly the chimpanzee parasite *Plasmodium reichenowi*[10,11] and are not present in other plasmodial species [2,12-15]. However, the genomes of these species of malarial parasites also contain multi-gene families thought to encode iRBC surface proteins, although the function of these proteins remains largely unknown. It is thought that altering expression of members of a multi-gene family functions as an immune evasion strategy while maintaining the essential function of the encoded protein. The *Plasmodium* Interspersed Repeats (PIR) multi-gene family is one such family that is well conserved in the human malaria parasite *Plasmodium vivax* (*vir*, n = 245), the monkey parasite *Plasmodium knowlesi* (*kir*, n = 68) and the rodent malaria parasites *Plasmodium berghei* (*bir*, n = 245), *Plasmodium chabaudi* (*cir*, n = 135) and *Plasmodium yoelii* (*yir*, n = 838) [12,14-17]. Unlike the clonal expression of *P. falciparum var* genes [18,19], many *pir* genes are concurrently transcribed during blood-stage infection [20-22], and in the case of the *yir* family, this expression appears relatively stable during a primary infection. Sequencing of the *P. yoelii* genome revealed another multi-gene family designated *pyst-a* (n = 140), which are homologous to *P. chabaudi* glutamate-rich proteins and to a single hypothetical protein in *P. falciparum* (PF14_0604 [3]), *P. vivax* (Pvx_117290, [3]) and *P. knowlesi* (PKH_124210, [3]). The glutamate-rich proteins of *P. chabaudi*, such as Pc90 (also known as Pc(em)93, Pc(em)96 and Pch105/RESA) are thought to localize to the cytoplasmic face of the RBC membrane and have been shown to be immunogenic [23-29]. To date, the function of the PYST-A family of proteins during blood-stage infection in *P. yoelii* has not been studied.

Rodent models of malaria present a unique opportunity to examine patterns of gene expression in vivo, allowing for use of cloned parasite strains replicating in inbred mice. These conditions may reduce some variability seen when using clinical isolates of *P. falciparum* parasites and are technically much more manageable. These in vivo model systems also allow for examination of gene expression at various time points post-infection and make it possible to manipulate the host immune system under controlled conditions. In this study, the *P.yoelii* 17X murine model was utilized to analyse how parasite gene expression patterns differ between individual animals infected with the same cloned parasite and how expression may change across independent infections initiated with different parasite populations. Gene expression at various time points post-infection and in the presence and absence of host immune pressure was also analyzed. A genome wide analysis was the primary focus but *yir* and *pyst-a* gene expression under these conditions was also examined. Results indicate that *P. yoelii* gene expression was remarkably stable supporting the notion that in vivo, external stressors and environmental factors have limited influence on gene transcription in *Plasmodium* blood-stage parasites.

## Methods

### Ethics statement

All animal studies were reviewed, approved and conducted in compliance with the Institutional Animal Care and Use Committee (IACUC) of Drexel University College of Medicine (protocol approval ID #18874). This IACUC operates with Public Health Service approval (Animal Welfare Assurance Number: A-322-01).

### Mice and parasites

Five to six week old male BALB/cByJ mice were purchased from The Jackson Laboratory (Bar Harbor, Maine, USA). Male BALB/cJ and B-cell-deficient J_H_D mice [30] on a BALB/cJ background were purchased from Taconic Farms Inc. (Germantown, NY, USA). All animals were housed in the Animal Care Facility of Drexel University College of Medicine under specific pathogen-free conditions. Food and water were provided ad libitum and the room was maintained on a 12-hour light–dark cycle. The lethal and non-lethal 17XL and 17X strains of *P. yoelii* were originally obtained from Dr William P Weidanz (University of Wisconsin, Madison, WI, USA) and maintained as cryopreserved stabilates. Blood from *P. yoelii* -infected mice contains a mixture of ring, trophozoite and schizont stage parasites as replication is asynchronous. During infection with these *P. yoelii* lines, gametocytes are not observed and parasitized blood is not infective for mosquitoes.

To establish cloned lines of *P. yoelii* 17X, groups of three to four male BALB/cByJ mice were infected i.v. with 25, 10 or one *P. yoelii* 17X iRBC(s). Blood parasitaemia was monitored by the enumeration of parasitized erythrocytes in thin tail-blood smears stained with Giemsa (EMD Chemicals, Inc., Gibbstown, NJ, USA). Parasites from the lowest infective dose were selected as clones.

### *Py* RMP preparation and generation of anti-*Py* RMP sera

Male BALB/cByJ mice were infected with 1x10^6^*P. yoelii* 17X iRBCs and blood was obtained when parasitaemia was ~30%. Infected RBCs were separated from uninfected RBCs by density gradient centrifugation on a 70% Percoll gradient (GE Healthcare, Uppsala, Sweden). Recovered iRBCs were treated with PBS-0.05% saponin and erythrocyte membranes were separated from intact parasites by differential centrifugation. Membranes from uninfected RBCs were also isolated as a control. Protein concentrations were determined using the bicinchoninic acid protein assay (Pierce Chemical Company, Rockford, IL, USA). The *P. yoelii* reticulocyte membrane protein (*Py* RMP) fraction, uninfected membrane proteins and parasite-associated antigen were separated by SDS-PAGE (15 μg/lane) on a 10% polyacrylamide gel and stained with Coomassie Blue. For the generation of polyclonal anti-*Py* RMP sera, mice were immunized subcutaneously three times at three-week intervals with 50 μg/dose of the *Py* RMP preparation formulated with 25 μg Quil A adjuvant (Accurate Chemical and Scientific Corporation, Westbury, NY, USA). Two weeks following the third immunization, sera was collected.

### Immunoblot analysis

The *Py* RMP fraction, uninfected membrane proteins and parasite-associated antigen were separated by SDS-PAGE as above and electroblotted onto nitrocellulose membranes. Membranes were then blocked with 5% (w/v) non-fat milk in TBS (25 mM Tris–HCl, pH 8.0, 150 mM NaCl) and probed with normal rabbit serum (1:20,000), polyclonal rabbit antiserum raised against recombinant *P. yoelii* MSP-8 (1:20,000), normal mouse serum (1:1000), or mouse anti-*Py* RMP (1:1000) diluted in TBS containing 0.1% (v/v) Tween 20 and 1% (w/v) BSA. Bound antibodies were detected by chemiluminescence using horseradish peroxidase conjugated protein A (Pierce Chemical Company) or rabbit anti-mouse IgG (Invitrogen, Carlsbad, CA, USA) and the SuperSignal West Pico Substrate (Pierce Chemical Company).

### Indirect-immunofluorescence assay

Blood was collected from *P. yoelii* 17X infected mice, washed and resuspended at ~25% haematocrit in PBS containing 1% (w/v) gelatin. Thin blood films were prepared, air dried and fixed in acetone:methanol (1:1) for 20 min at −20°C. Fixed smears were incubated for 30 min at 37°C in a humidified chamber with anti-*Py* RMP sera or normal mouse sera diluted 1:200 in PBS. Bound antibody was detected using tetramethyl rhodamine (TRITC)-conjugated goat anti-mouse immunoglobulin G (IgG) (Invitrogen) diluted 1:250 in PBS. Slides were stained with Hoechst 33258 diluted 1:47,000 in 1X PBS, washed, and mounted with AntiFade reagent (Invitrogen Molecular Probes, Eugene OR, USA). Images (1,000X magnification) were obtained using an Olympus BX60 fluorescent microscope (Olympus America Inc., Melville, NY, USA) and a SPOT RT Slider Digital Camera System (Diagnostic Instruments, Sterling Heights, MI, USA).

### Immunizations and infections

Normal and immunodeficient naïve mice were infected by intraperitoneal injection of 1x10^5^*P. yoelii* 17X infected RBCs. Infected RBCs for RNA isolation were obtained early in infection when parasitaemia was ~15% (day 10–11), at peak parasitaemia (~35-40%, day 14) or during descending parasitaemia (~15%, day 18). Alternatively, mice were immunized subcutaneously three times at three week intervals with 50 μg of the *Py* RMP fraction formulated with 25 μg of Quil A adjuvant. Control groups received Quil A alone. Two weeks following the final immunization, animals were challenged with 1x10^5^*P. yoelii* 17X iRBCs. Infected RBCs for RNA isolation were obtained from *Py* RMP-immunized and adjuvant control mice on day 10–12 of infection.

### *Plasmodium yoelii* DNA microarrays

*Plasmodium yoelii* DNA microarrays were produced in the Molecular Genomics Core Facility, Drexel University College of Medicine, under the direction of LWB. Each array contained 65-base oligonucleotides, spotted in duplicate, representing ~6,700 coding regions predicted from the *P. yoelii* genome sequence analysis [12] and present in the current PlasmoDB and NCBI databases. Each quadrant of an array contained a pool of *P. yoelii* oligos (~2200), spotted in triplicate, which served as a positive control. Blood from *P. yoelii* -infected mice was obtained from individual animals or pooled from groups of animals (five to 10) as needed. Infected RBCs were isolated as above by Percoll density gradient centrifugation. Infected RBCs were saponin lysed and pelleted *P. yoelii* parasites were resuspended directly in the TRIzol Reagent (Invitrogen). Total RNA was extracted, precipitated and purified using an RNeasy RNA isolation kit (Qiagen, Inc., Valencia, CA, USA).

For all microarrays, gene expression in *P. yoelii* 17X blood-stage parasites was evaluated relative to a standard comparator of purified *P. yoelii* 17XL total RNA. *P. yoelii* RNA (5 μg/sample) was amplified in the presence of aminoallyl-dUTP using the Amino Allyl MessageAmp II aRNA amplification kit (Ambion, Inc., Austin, TX, USA). aRNA was then fluorescently labelled by reaction with monofunctional, NHS-activated Cy3 or Cy5 dyes (Amersham Biosciences Inc., Piscataway, NJ, USA). Cy dye labelled aRNA was purified with yield and specific activity of each probe determined by absorption spectroscopy. Pairs of Cy3 and Cy5 labelled aRNA probes (1 μg/probe) were pooled, fragmented (Ambion RNA Fragmentation Reagent) and hybridized to the *P. yoelii* microarrays for 14–16 hours at 65°C. Following hybridization and washing, slides were scanned using a GenePix 4000A microarray laser scanner (Axon Instruments, Inc., Union City, CA, USA) and the fluorescence intensity of each DNA feature was determined at 532 nm (Cy3) and 635 nm (Cy5). Data for each gene was obtained from replicate features on each array. In addition, replicate arrays and standard dye flips were also hybridized for each comparison. Fluorescence data were acquired and initially analysed using GenePixPro 5.1 Software (Axon Instruments, Inc.). Irregular and missing features flagged during image acquisition, features with a diameter of ≤60 μm and features with a signal to noise ratio (SNR) of <2 on the 532 nm channel and SNR <2 on the 635 nm channel were removed from the analysis.

### Data analysis

In order to evaluate differential gene expression in *P. yoelii* 17X parasites replicating in various in vivo environments, Cy3 and Cy5 fluorescence intensity data were processed and analysed using limma version 2.18.2 [31,32] through limmaGUI version 1.20.0 [33] in the R programming environment [34] The normexp method with an offset value of 16 was utilized for background correction [35,36]. Data were normalized using the print-tip loess method [37]. After fit of a linear model to the expression data, genes differentially expressed by at least two-fold were identified (log2-fold change >1 or < −1). Such changes in gene expression were considered significant based on a *p* -value ≤ 0.01 obtained from the moderated t-statistic and adjusted for multiple testing using the method of Benjamini and Hochberg [38]. The false discovery rate was 1%. An initial data analysis revealed relatively small numbers of differentially expressed genes across conditions. As such, the final statistical analysis was completed by multiple pairwise comparisons of groups, accepting the increased possibility of a type I error - erroneously detecting differences in gene expression.

The number of *yir* and *pyst-a* genes expressed in a given population of *P. yoelii* 17X parasites was estimated based on normalized signal intensity relative to the entire *P. yoelii* gene set. Fluorescence data were captured and filtered as described above and analysed using the Acuity 3.1 Microarray Informatics Software package (Axon instruments, Inc). On each array, Cy3 and Cy5 signal intensities were normalized such that the ratio of the median fluorescence intensity of 192 positive control features equalled 1. A normalized fluorescence intensity for all genes was calculated by setting the fluorescence intensity of the positive control to 3,000. Mean fluorescence intensity (MFI) was then calculated for each gene across an array set (i.e., replicate arrays, dye swaps) and percentiled. Based on mapping data available through PlasmoDB, 464/859 *yir* gene oligos and 74/140 *pyst-a* gene oligos on the arrays detect unique transcripts (Additional file 1). Non-discriminating *yir* and *pyst-a* oligos were eliminated from the analysis. Expression of ‘unique’ genes was categorized as below detection (0-50^th^ percentile), low (51-75^th^ percentile), moderate (76-90^th^ percentile) or high (>90^th^ percentile).

### *Plasmodium yoelii* gene/protein categories

**Figure 1:**
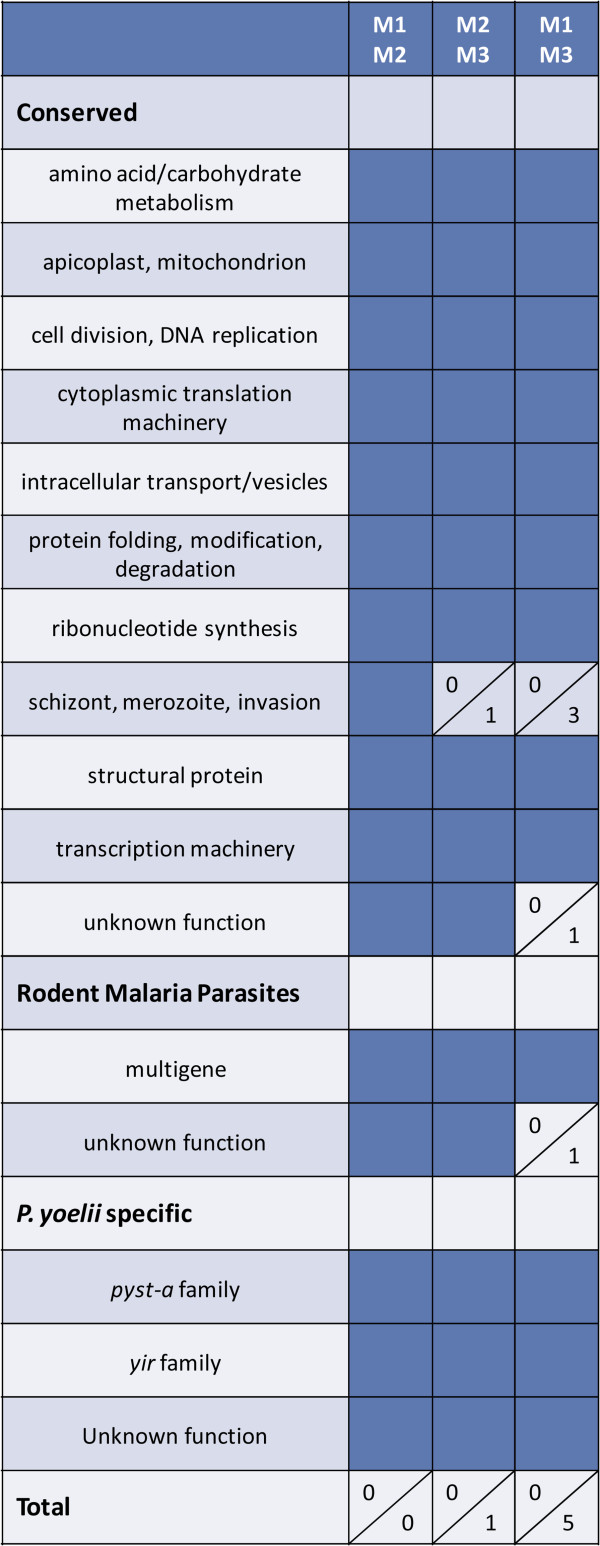
**There is little differential gene expression between *Plasmodium yoelii *17X parasites isolated from individual mice.** Three animals (M1, M2 and M3) were infected with *P. yoelii* 17X iRBCs from a single donor mouse. RNA was isolated on day 10 or 11 post-infection and transcripts detected using *P. yoelii* DNA microarrays. The three possible pair-wise comparisons are listed at the top of the figure and functional categories are listed in the far left column. Solid dark blue boxes indicate that there are no differentially expressed genes for a given comparison in a particular functional group. In cases where there are differentially expressed genes, the number of genes that are up-regulated is listed at the top half of the box; the number of down-regulated genes is listed in the bottom half of the box. Up- and down- regulation is reported with respect to the second animal listed in the column heading. For example, there is one gene down-regulated in the ‘schizont, merozoite, invasion’ category in mouse 3 with respect to mouse 2. The total number of genes that were up- or down-regulated is indicated at the bottom of the Figure.

In an attempt to determine if the expression of genes involved in similar processes changes in a coordinated manner in response to the in vivo environment, *P. yoelii* genes were grouped into several categories. Bioinformatic data available from the sequencing and annotation of the *P. yoelii* genome were considered. As this data set is incomplete, a substantial set of data available on orthologs of *P. yoelii* genes present in the genomes of *P. falciparum*, *P. vivax*, *P. knowlesi*, *P. berghei* and *P. chabaudi* were consulted [3]. Likewise, published comparative genomic data and in depth transcriptional profiling data were also considered. These included the analysis of the *P. falciparum* transcriptome of in vitro cultured *P. falciparum* blood-stage parasites [4], the analysis of changes in *P. falciparum* gene expression in cultured blood-stage parasites upon exposure to various chemical compounds [39] and genome-wide comparisons of *P. yoelii*, *P. berghei* and *P. chabaudi*[40]. Using these resources, differentially expressed *P. yoelii* genes were grouped into three broad categories: i) genes conserved across malarial species; ii) genes specific to the rodent malarial parasites; and iii) genes that appear to be unique to *P. yoelii* . Within these broad categories, differentially expressed genes were assigned to 16 subcategories (see Figure 1 and Additional file 2). The evaluation of genes encoding mitochondria-associated proteins (Additional file 3) included the set of *P. yoelii* orthologs of putative *P. falciparum* mitochondrial proteins [7,41] (Mather and Vaidya, unpublished data) for which signal intensity across arrays was consistently above the 50^th^ percentile. 

### Data deposition

The microarray data reported in this paper have been deposited in the Gene Expression Omnibus database (GEO) (accession no. GSE31274) [42]. Deposited data are MIAME compliant [43,44].

## Results

### Little variation in gene expression in *Plasmodium yoelii* 17X blood-stage parasites obtained from individual mice challenged with the same inoculum of iRBCs

**Figure 2:**
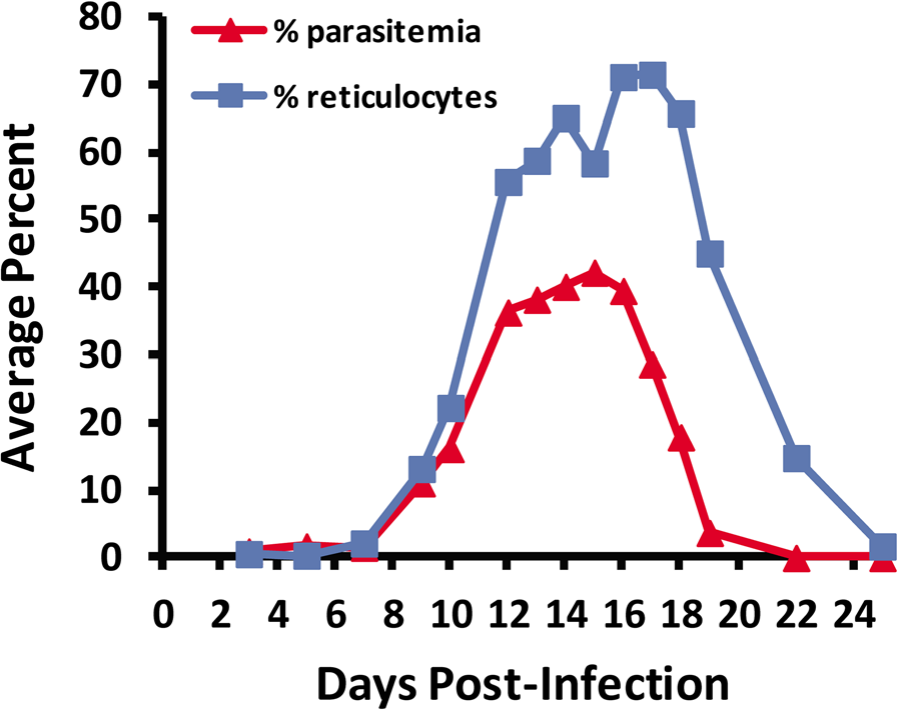
**Course of *Plasmodium yoelii *17X blood stage infection in male BALB/cByJ mice.** Animals (n = 5) were infected by intraperitoneal injection with 1x10^5^*P. yoelii* 17X infected RBCs. Parasitaemia and percent reticulocytes were monitored in thin tail-blood smears stained with Giemsa. Average percent parasitaemia (red) and reticulocytes (blue) at various days post-infection are shown.

In the *P. yoelii* 17X model, peak parasitaemia generally occurs on day 14–16, reaching parasitaemias of 40-45% in BALB/c mice. During this time, the primary immune response that ultimately leads to parasite clearance develops. The percentage of reticulocytes peaks at ~70% slightly later post-infection (days 16–18). Parasites are generally cleared from the circulation by day 22–25 post-infection (Figure 2). As a starting point in evaluating variability in gene expression in blood-stage parasites replicating in vivo in different hosts, three mice were simultaneously infected with 1x10^5^*P. yoelii* 17X iRBCs from a single donor mouse. Parasite diversity was minimized by using a cloned *P. yoelii* 17X line. Host diversity was minimized by using age-matched, male, inbred BALB/cByJ mice housed together. Parasite RNA was isolated early during infection when parasitaemia was ascending as follows: mouse 1 - day 11 at 13.0% parasitaemia; mouse 2 - day 10 at 11.1% parasitaemia; mouse 3 - day 10 at 18.6% parasitaemia. Using *P. yoelii* DNA microarrays (~6,700 oligos), gene expression profiles were then compared between individual mice with three pair-wise comparisons (M1/M2, M2/M3 and M1/M3). In all comparisons, a change in expression of at least two-fold (*p* ≤0.01) was considered significant. As shown in Figure 1 and Additional file 2, gene expression profiles between individual mice were remarkably consistent. There were no differentially expressed genes between mouse 1 and mouse 2. Only one gene, merozoite surface protein-1 (MSP1), was down-regulated ~2.3-fold in mouse 3 relative to mouse 2. There were only five genes differentially expressed when comparing mouse 1 and mouse 3 which included MSP1, two rhoptry proteins and two proteins with unknown function. As such, there were minimal differences in gene expression profiles of *P. yoelii* 17X parasites isolated from three separate hosts after a 10–11day period of replication in vivo.

**Figure 3:**
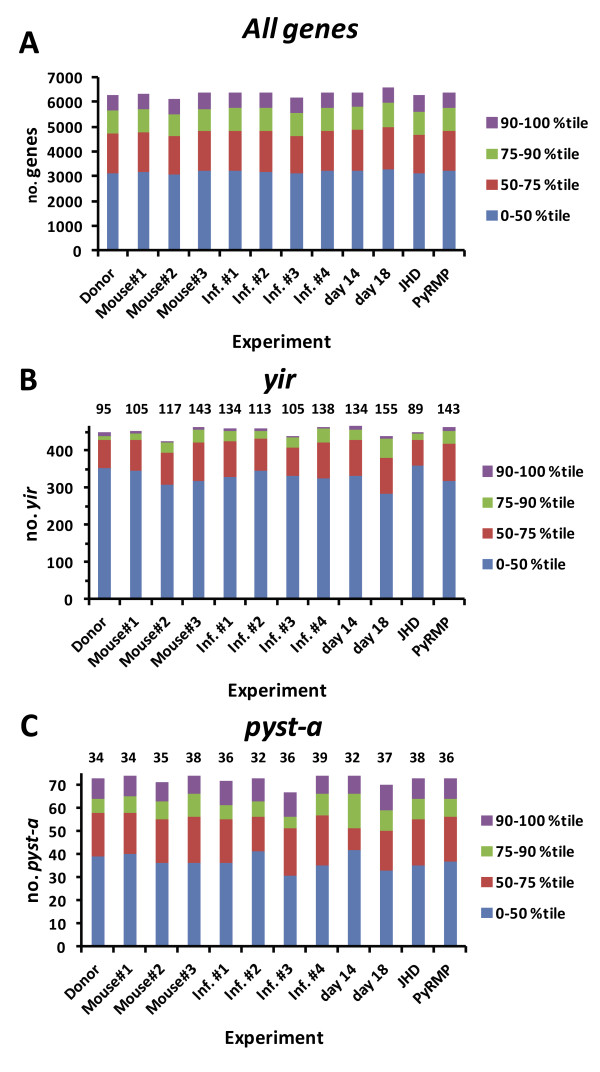
***Plasmodium yoelii *17X blood-stage parasites express a large number of *yir *and *pyst-a *during infection.** Animals were infected with *P. yoelii* 17X iRBCs and parasite RNA was collected under various experimental conditions (see Methods). *P. yoelii* DNA microarrays were completed and a normalized mean fluorescence intensity for all genes spotted on the *P. yoelii* array was calculated and percentiled (**A**). Number of *yir* (**B**) and *pyst-a* (**C**) genes in the listed percentile ranges were calculated considering a set of 464 *yir* gene oligos and 74 *pyst-a* oligos that could detect unique transcripts. The number of *yir* or *pyst-a* genes expressed (i.e., those with a mean fluorescence intensity above the 50^th^ percentile) is listed at the top of the Figure above each experimental condition.

In the above analysis, no members of the *yir* or *pyst-a* multigene families were differentially expressed between individual animals. To estimate the number of *yir* and *pyst-a* genes expressed in each population of *P. yoelii* 17X parasites, signal intensity on each set of arrays was normalized and percentiled relative to the entire *P. yoelii* gene set (Figure 3A). Focusing the analysis on a set of 464 *yir* gene oligos that could detect unique transcripts, expression (>50^th^ percentile) of a relatively large number of *yir* genes (105, 117, 143) was measurable in parasites isolated from the three individual mice (Figure 3B). Combined, 20–40 of these were considered to be expressed at moderate (76-90^th^ percentile) or high (>90^th^ percentile) levels. Likewise, based on data obtained with a subset of 74 unique oligos, expression (>50^th^ percentile) of multiple *pyst-a* genes (34, 35, 38) was observed in the three populations of *P. yoelii* 17X parasites, with 16–18 detected at moderate to high levels (Figure 3C). Differences in the number of ‘expressed’ *yir* and *pyst-a* genes in each group are due to a small number of genes whose signal intensity was near the threshold set for detection (50^th^ percentile) but where fold-change was less than two and/or not statistically significant.

### Differential gene expression in *Plasmodium yoelii* 17X blood-stage parasites marginally increases over time

**Figure 4:**
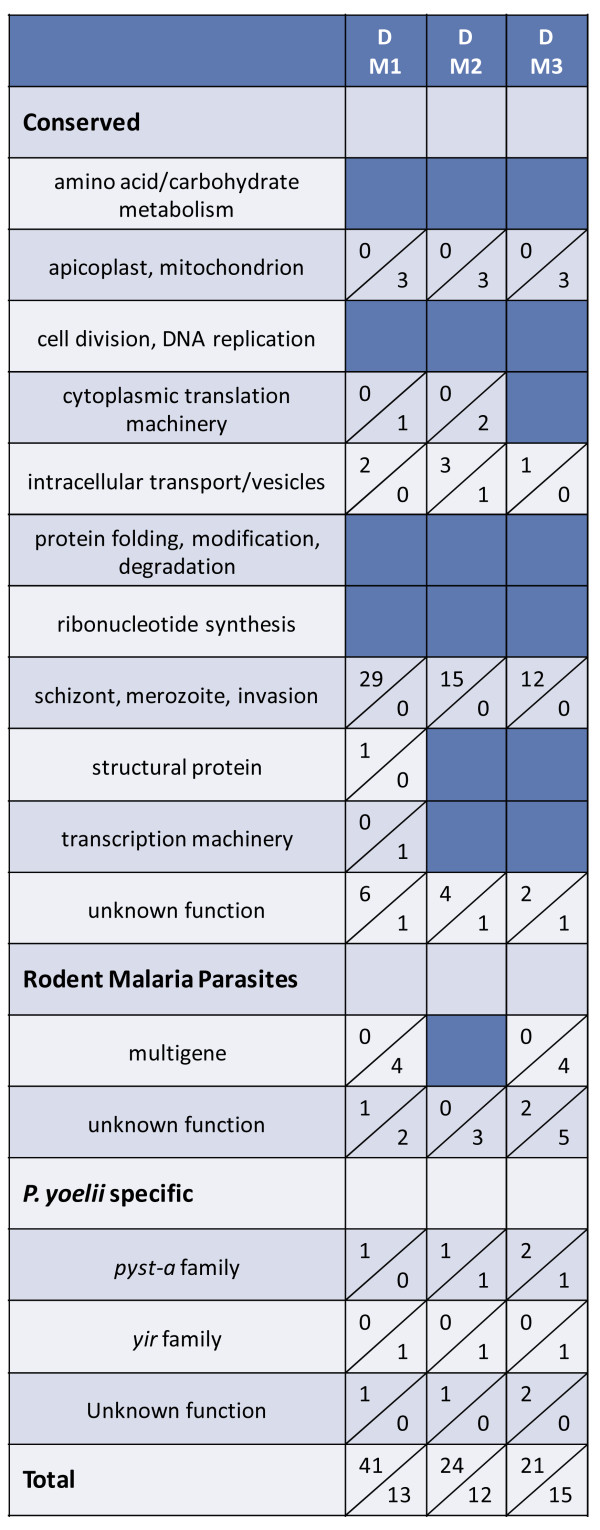
**Modest increase in differential gene expression over the course of a primary *Plasmodium yoelii *17X infection.** Three animals (M1, M2 and M3) were infected with *P. yoelii* 17X iRBCs from a single donor mouse (D). RNA was isolated on day 10 or 11 post-infection and *P. yoelii* DNA microarrays were completed. The three pair-wise comparisons between individual mice and the donor are listed at the top of the Figure, and functional categories are listed in the far left column. Up- and down-regulation for each comparison is indicated as described for Figure 1.

To determine if *P. yoelii* gene expression profiles changed significantly over time in vivo, the patterns of gene expression in the three infected mice described in Figure 1 above (day 10–11) were compared to that of the population of *P. yoelii* 17X parasites isolated from the donor animal used to initiate infection on day 0. The donor mouse was infected using cryopreserved *P. yoelii* 17X iRBCs. On day 11 of infection in this donor mouse when parasitaemia was 18.7%, *P. yoelii* 17X iRBCs were isolated for infection of M1, M2 and M3 and for RNA isolation. As shown in Figure 4 and Additional file 4, some variation in expression profiles was noted in three pair-wise comparisons between the donor (D) and each infected mouse (M1, M2, M3). Overall however, the number of differentially expressed genes remained relatively low as the expression of only 54, 36 and 36 genes was significantly altered in M1, M2 and M3 relative to the donor mouse, respectively. The magnitude of the changes ranged from 2 to 5.8-fold. Genes associated with schizont rupture and/or merozoite invasion of RBCs were consistently up-regulated in mouse 1, 2 and 3 relative to the donor animal, and included *P. yoelii* orthologs of several rhoptry proteins, MSP1, MyoA, MTIP, SERA and subtilisin-like protease 2. Similar to the comparisons between individual mice, expression of a fairly large number of the *yir* (95/464) and *pyst-a* (34/74) genes was detected above background (>50^th^ percentile) in the donor mouse (Figure 3). Changes in *yir* and *pyst-a* gene expression from day 0 in the donor mouse to day 10–11 in the three infected mice were not remarkable.

### Gene expression profiles in *Plasmodium yoelii* 17X blood-stage parasites remain relatively stable in independently infected mice

**Figure 5:**
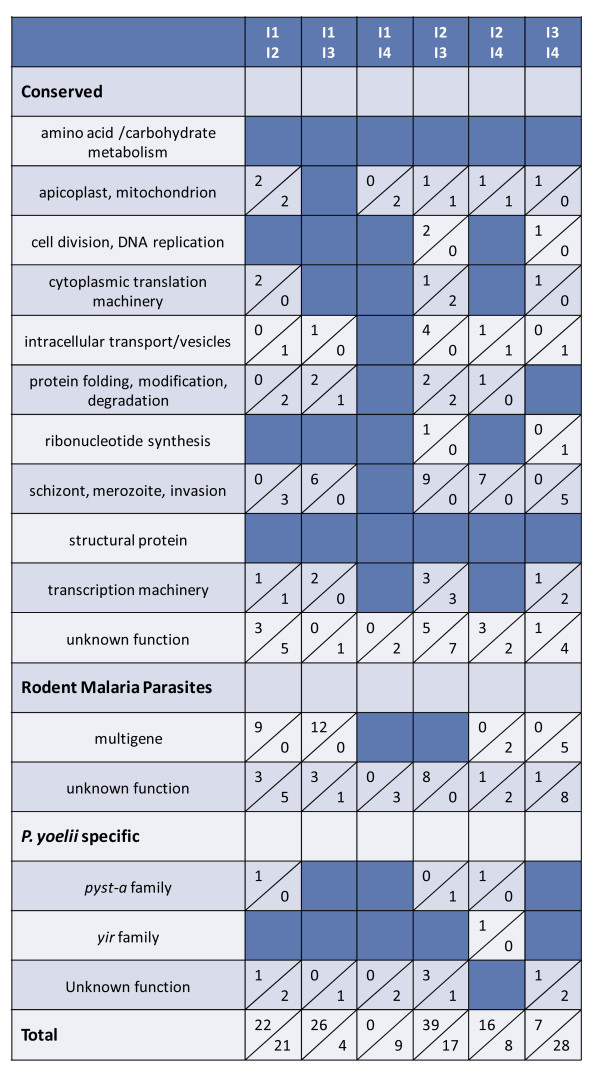
**Differential gene expression is lower than expected in parasites from independent *Plasmodium yoelii *17X infections.** Groups of animals (n = 5-10) were infected on four separate occasions (I1, I2, I3 and I4) using four separate donor animals. RNA was isolated and transcripts detected using *P. yoelii* DNA microarrays. The six possible pair-wise comparisons are listed at the top of the Figure and functional categories are listed in the far left column. Up- and down-regulation for each comparison is indicated as described for Figure 1.

Given the relatively few differences noted between donor and individual infected mice, it was of interest to examine variability in *P. yoelii* 17X gene expression across independently initiated infections (I1, I2, I3, I4) in animals housed separately in time and space. On four separate occasions, blood from a donor mouse was used to infect groups of BALB/cByJ mice (n=10). For each infection, blood from *P. yoelii* 17X infected mice was obtained and pooled on days 10–11 of infection when parasitaemia was ascending and was ~15%. *P. yoelii* 17X RNA from these four independent infections permitted six pair-wise comparisons. Gene expression profiles varied to a greater degree between these independently initiated infections (Figure 5 and Additional file 5) than between individual animals concurrently infected with the same inoculum of *P. yoelii* 17X iRBCs (Figure 1 and Additional file 2). Nevertheless, differences were again lower than expected with the number of genes differentially expressed between any two infections ranging from only 9 to 56 genes. In each case, at least 40% of differentially expressed genes are predicted to encode proteins of unknown function. The remaining genes in each set were associated with multiple and diverse functions. Consistent with the comparisons thus far, each population of *P. yoelii* 17X blood-stage parasites expressed a relatively large number of *yir* genes (105 to 138) and *pyst-a* genes (32 to 39) over background (>50^th^ percentile) (Figure 3).

### *Plasmodium yoelii* gene expression is influenced by parasitaemia and/or reticulocyte levels

**Figure 6:**
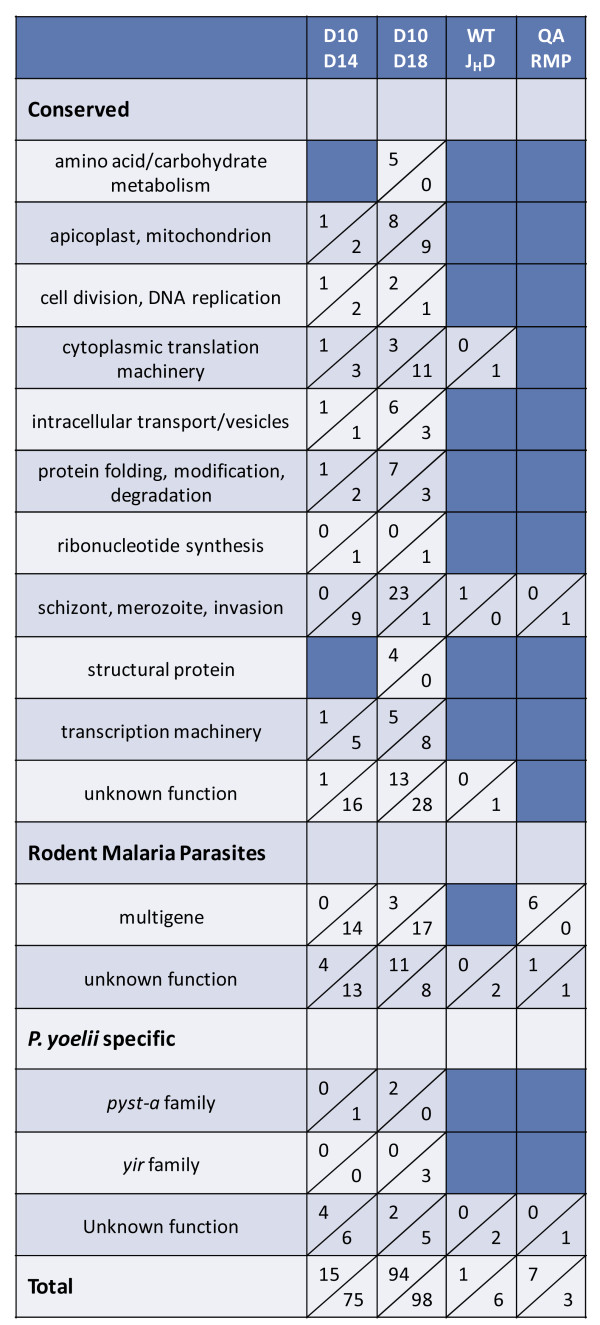
**Parasitaemia and/or host cell availability influence differential gene expression of *Plasmodium yoelii *17X parasites.** Groups of wild-type animals (n=5-10) were infected with *P. yoelii* 17X iRBCs and parasite RNA was isolated on day 10, day 14 or day 18 post-infection. Immunologically intact (WT) and B-cell deficient J_H_D animals were similarly infected and parasite RNA was isolated on day 10. A third set of mice was immunized three times with a preparation of *P. yoelii* reticulocyte membrane proteins ( *Py* RMP) plus QuilA as adjuvant (RMP) or with Quil A alone (QA) prior to *P. yoelii* challenge. Parasite RNA was isolated on day 10 (QA) or day 12 (RMP) post-infection. The number of genes up- or down-regulated for a given comparison and category is listed as described in Figure 1.

Thus far, *P. yoelii* gene expression was measured early during acute infection and at consistent parasitaemia. However, *P. yoelii* 17X preferentially invades reticulocytes and at this early point in infection, the number of reticulocytes in circulation is low (~15-20%). A sharp increase in erythropoiesis leads to a marked influx of new reticulocytes into peripheral circulation and a concurrent rise in *P. yoelii* 17X parasitaemia. It was of interest to determine if changes in parasite load and permissive host cell availability affected *P. yoelii* gene expression in vivo. *P. yoelii* 17X iRBCs from a single donor mouse were used to infect a set of BALB/cByJ mice. RNA was isolated from *P. yoelii* iRBCs harvested on day 10 (14% parasitaemia, 14.4% reticulocytes) and on day 14 (43.5% parasitaemia, 57.9% reticulocytes) of infection. Comparing *P. yoelii* 17X gene expression profiles, there were 15 genes significantly up-regulated and 75 genes significantly down-regulated on day 14 relative to day 10 (Figure 6 and Additional file 6). Overall, the magnitude of the changes in gene expression was again modest varying only 2 to 5.7-fold. Putative protein functions appeared to be diverse, with genes encoding proteins of unknown function being the most commonly represented group. These tended to be down-regulated on day 14 relative to day 10. In addition and despite an overall increase in the number of differentially expressed genes, the expression of *yir* and *pyst-a* gene families remained relatively constant (Figures 3 and 6).

To further evaluate changes in parasite gene expression during the course of infection, *P. yoelii* 17X RNA was isolated on day 10 (19% parasitaemia, 18.6% reticulocytes) and on day 18 (14% parasitaemia, 70.2% reticulocytes). Although parasite load is similar at these time points, parasitaemia is ascending on day 10 and target host cells are relatively scarce. By day 18 post-infection, parasitaemia is declining due to immune-mediated mechanisms and reticulocytes are abundant. Comparing day 10 and day 18, there were 192 differentially expressed *P. yoelii* genes, including 67 genes of unknown function (Figure 6 and Additional file 6). Of those differentially expressed, 94 were up-regulated and 98 were down-regulated on day 18 versus day 10. This set of differentially expressed genes represents the most variable expression seen across all experimental conditions. Of interest, genes encoding proteins associated with the cytoplasmic translation machinery tended to be down-regulated on day 18 (n = 11) versus day 10, and included several ribosomal proteins. In addition, a small set of genes (n = 24) encoding proteins involved with schizont rupture and/or merozoite invasion of RBCs tended to be up-regulated on day 18 and these included glidosome- and rhoptry-associated genes, MSP1 and MSP7. Finally, there were 17 genes encoding apicoplast/mitochondrion-associated proteins that were differentially expressed, with up and down-regulated genes equally represented. As before, members the *yir* and *pyst-a* multigene families were not differentially expressed while the number of genes in each family expressed on day 18 remained stable (Figure 3, 155 *yir* genes, 37 *pyst-a* genes).

### Changes in immune pressure do not significantly alter *Plasmodium yoelii* 17X gene expression during a primary infection

To specifically evaluate whether the presence or absence of host immune pressure affects parasite gene expression *in vivo*, two experimental approaches were taken. In the first approach, all antibody-dependent immune pressure was removed by use of B cell deficient mice. Naïve, immunocompetent BALB/c mice and B cell deficient, J_H_D mice on a BALB/c background were concurrently infected with *P. yoelii* 17X iRBCs obtained from the same donor animal. *P. yoelii* gene expression in the two hosts was evaluated when ascending parasitaemia reached ~15%. Surprisingly, changes in gene expression were negligible with only seven differentially expressed genes, five of which were of unknown function (Figure 6 and Additional file 6). No *yir* or *pyst-a* genes were differentially expressed between the populations of *P. yoelii* 17X parasites.

**Figure 7:**
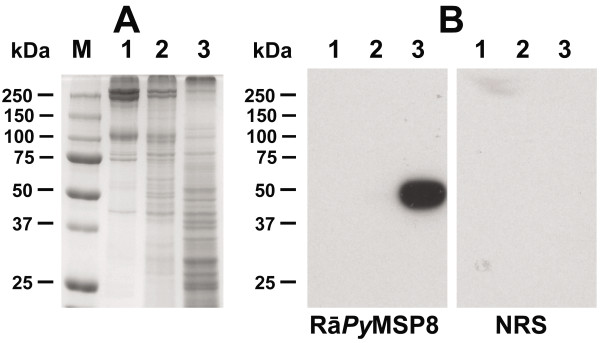
**Membrane preparations from *Plasmodium yoelii*-infected RBCs contain unique parasite proteins.** Animals were infected with *P. yoelii* 17X and infected RBCs were collected. (**A**). Proteins associated with the membranes of uninfected (lane 1) or *P. yoelii* 17X infected erythrocytes (lane 2) or with intracellular *P. yoelii* 17X parasites (lane 3) were separated by SDS-PAGE and stained with Coomassie Blue. (**B**). To evaluate the ability to separate the *Py* RMPs from parasite associated proteins, isolated protein fractions were separated as in A, blotted onto nitrocellulose and probed with rabbit anti-mouse *Py* MSP8 polyclonal sera. *Py* MSP8 is a GPI-anchored membrane protein of *P. yoelii* trophozoites and merozoites. Normal rabbit sera (NRS) served as a negative control. Molecular weights in kiloDaltons (kDa) are indicated.

**Figure 8:**
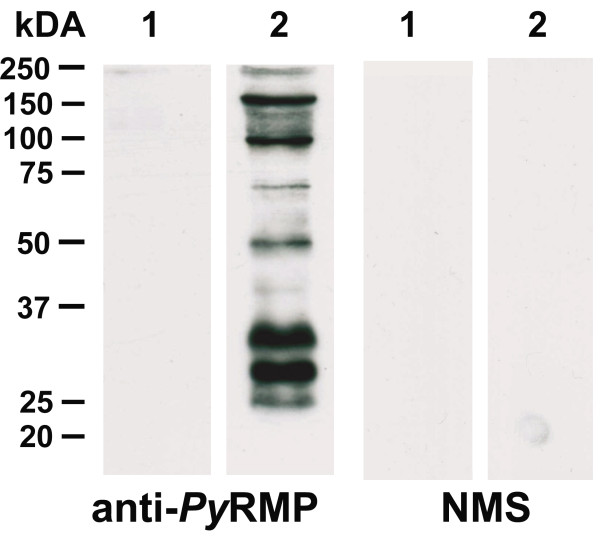
**Immunization induces antibody responses to proteins associated with the *Py*RMP fraction.** Animals were immunized three times with the *Py* RMP preparation and sera were collected. Proteins associated with the membrane of uninfected RBCs (lane 1) or *P. yoelii* 17X infected RBCs (lane 2) were separated by SDS-PAGE, immunoblotted and probed with a pool of *Py* RMP antisera. Normal mouse sera (NMS) served as a negative control. Molecular weights in kiloDaltons (kDa) are indicated.

**Figure 9:**
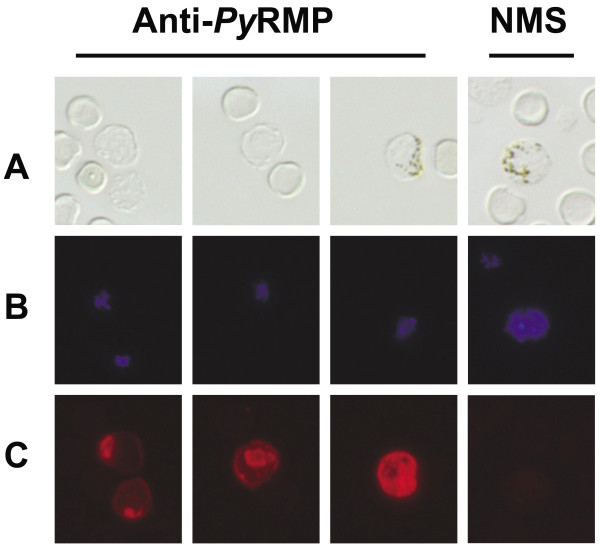
**Anti-*Py*RMP antibodies detect *Plasmodium yoelii *proteins associated with the intracellular parasite and the reticulocyte membrane.** Immunofluorescence of *P. yoelii* 17X infected reticulocytes using polyclonal *Py* RMP sera or normal mouse sera. Row A: Differential interference contrast images; Row B: Parasite DNA stained with DAPI (blue); Row C: Localization of *P. yoelii* proteins recognized by anti-*Py* RMP sera (TRITC, red). Background staining with normal mouse sera (NMS) is shown.

**Figure 10:**
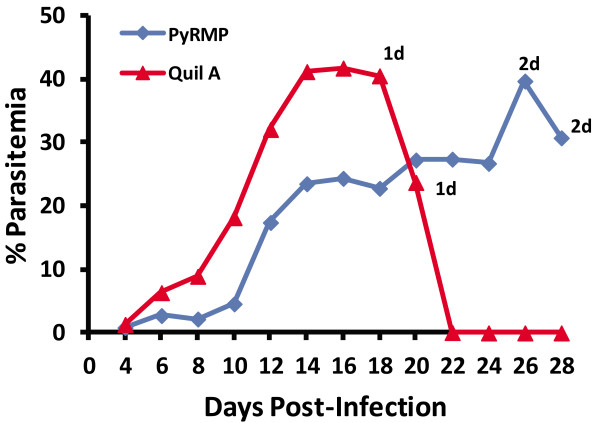
**Course of *Plasmodium yoelii *17X infection following *Py*RMP immunization.** Animals (n = 5) were immunized three times with the *Py* RMP preparation plus Quil A adjuvant. A separate group of animals received Quil A alone. Both groups were challenged with 1x10^5^*P. yoelii* 17X iRBCs intraperitoneally. Parasitaemia was monitored in thin tail-blood smears stained with Giemsa at various time points post-infection. ‘#d’ indicates the number of animals at each time point that were sacrificed due to high parasitaemia and/or significant anemia.

In a second approach, antibody-mediated immune pressure was applied by immunizing animals with a preparation of membrane proteins isolated from *P. yoelii* 17X infected reticulocytes (*P. yoelii* reticulocyte membrane proteins, *Py* RMPs). SDS-PAGE and Coomassie-blue staining of proteins associated with the membranes of uninfected versus *P. yoelii* 17X infected RBCs revealed several protein bands unique to the *Py* RMP fraction (Figure 7A, lanes 1 & 2). Immunoblot analysis to assess separation of reticulocyte membrane proteins from parasite membrane proteins showed that the GPI-anchored merozoite surface protein 8 was present in parasite but not reticulocyte membrane fractions (Figure 7B). Antibodies from mice immunized with the *Py* RMP preparation recognized 8–10 distinct polypeptides present in the *Py* RMP fraction (Figure 8). In indirect immunofluorescence assays, these antibodies specifically stained proteins localized to both the intracellular parasite and the iRBC membrane (Figure 9). To determine if the presence of *Py* RMP-specific antibodies affected parasite growth and gene expression, *Py* RMP immunized and adjuvant control (Quil A) mice were challenged with *P. yoelii* 17X iRBCs from the same donor mouse. To some degree, *Py* RMP immunization limited parasite replication early during infection but over time these animals developed relatively high and persistent parasitaemia and were sacrificed (Figure 10). To determine if the presence of anti-*Py* RMP antibodies influenced patterns of gene expression, RNA was isolated from *P. yoelii* 17X parasites on day 10 from Quil A control mice (15.6% parasitaemia) or day 12 from *Py* RMP immunized mice (13.3% parasitaemia) post-infection and subjected to microarray analysis (Figure 6 and Additional file 6). Similar to the results in J_H_D mice in the absence of antibody responses, increasing immune pressure by *Py* RMP immunization led to negligible differences in gene expression. Comparison of expression profiles of *P. yoelii* 17X parasites isolated from Quil A versus *Py* RMP immunized mice revealed only 10 differentially expressed genes including three with no known function. Unexpectedly, no members of the *yir* or *pyst-a* multi-gene families were differentially expressed. Consistent with the earlier data however, expression (>50^th^ percentile) of 134 *yir* genes and 36 *pyst-a* genes in Quil A control mice and 143 *yir* genes and 36 genes *pyst-a* in *Py* RMP immunized mice was detected (Figure 3, Inf. #1 &*Py* RMP). Combined, the data indicate that in vivo, *P. yoelii* 17X gene expression was only minimally altered by increasing or decreasing antibody-mediated immune pressure.

## Discussion

Transcription in *P. falciparum* and *P. vivax* parasites appears to be tightly regulated, resulting in a ‘continuous cascade’ of gene expression during blood-stage development [4,5,45]. There is some evidence that this transcriptional programme continues unabated even in the presence of external stresses. In one study, anti-folate treatment of cultures of drug-sensitive *P. falciparum* blood-stage parasites did not lead to increased expression of the defined drug target, dihydrofolate reductase-thymidylate synthatase. Additionally, there were no genes differentially regulated greater than two-fold between control and drug treated cultures [46]. Similarly, Gunasekera *et al.*[47] found little variation in transcription patterns between untreated and chloroquine-treated *P* . *falciparum* parasites growing asynchronously in culture. These results were unexpected, as genome-wide studies in organisms such as *Saccharomyces cerevisiae*, *Mycobacterium tuberculosis* and *Candida albicans* revealed differential expression of hundreds of genes in response to drug treatment, and these changes often confirmed or revealed associated drug targets and affected pathways [48-51]. However, other studies in *Plasmodium* examining the effects of drug treatment or manipulation of culture conditions have found more substantial transcriptional changes (~300-400 differentially expressed genes), especially in genes predicted to encode proteins exported to the host cell cytoplasm and the RBC membrane [52,53]. The work shown here extends these efforts significantly by focusing on malaria parasites replicating in vivo, in a more complex setting. With *P. yoelii* 17X blood-stage parasites, global gene expression profiles were remarkably consistent even in the presence of a changing host environment.

Initially, three genetically identical, age-matched BALB/cByJ mice were infected with the same inoculum of *P. yoelii* 17X iRBCs obtained from a donor mouse. *P. yoelii* RNA was isolated from each animal after a 10–11 day period of replication in vivo. As the in vivo environment is expected to be comparable in this setting, only a small set of differentially expressed genes was anticipated. Parasite gene expression patterns in each of the three animals were virtually identical with only five to six differentially expressed genes. Between the initiation of infection and harvest of iRBCs for RNA isolation, *P. yoelii* parasites in this study completed 10–12 replication cycles in vivo. Over this time, the number of differentially expressed genes was expected to increase somewhat relative to the population of *P. yoelii* iRBCs used to initiate the infection. This was the case, but the increase was relatively small involving only 40–60 genes out of the set represented by ~ 6,700 oligonucleotides on the arrays. A small cluster of genes expressed in late-stage parasites and potentially involved in merozoite invasion of host cells were differentially expressed between the donor mouse and recipients. This may not be surprising as iRBCs were harvested early during ascending parasitaemia when *P. yoelii* 17X parasites can be found in varying degrees in both normocytes and reticulocytes. This change in gene expression may reflect utilization of multiple invasion pathways. Finally, a number of potentially influencing variables were further increased by comparing gene expression profiles of *P. yoelii* 17X blood-stage parasites harvested from four independent infections initiated with iRBCs obtained from separate donor mice. Remarkably, the number of differentially expressed genes in pair-wise comparisons remained low (10–50 genes). These represent genes that are involved in diverse or unknown functions and at present, the associated changes do not appear to reflect a biologically significant response to the host environment. This, combined with a false discovery rate of 1% associated with this analysis suggest that detection of a low number of differentially expressed but largely unrelated genes could simply represent ‘noise’ inherent to large microarray analysis.

Non-lethal *P. yoelii* 17X parasites replicate preferentially in reticulocytes but do invade and develop within mature RBCs when reticulocytes are limiting. Parasites harvested on day 10 of *P. yoelii* 17X infection will be present in both reticulocytes and normocytes. As a result of an influx of new reticulocytes into circulation, *P. yoelii* 17X parasites will be found almost exclusively in reticulocytes by day 14 post-infection. As such, substantial differences in patterns of *P. yoelii* 17X gene expression in parasites harvested on day 10 versus day 14 were expected. In addition to the shift in host cell tropism, the ongoing infection increases parasite burden, host stress and immune pressure that could also influence *P. yoelii* 17X expression profiles. While the number of differentially expressed genes in this comparison increased to 95, these were not clustered based on related function or biological process and the majority were of unknown function. In an early study, utilizing lethal *P. yoelii* 17XL parasites, a similar number of genes altered in association with a shift in host cell preference from normocytes to reticulocytes were identified [54]. The function of these genes will be revisited as the annotation of both *P. falciparum* and *P. yoelii* genomes progresses. To further examine the role of host cell availability and immune pressure, gene expression in parasites isolated on day 10 and day 18 of infection was compared. Parasitaemia at these two time points is comparable. However on day 18, 70-75% of RBCs in circulation are reticulocytes and *P. yoelii* iRBCs are rapidly being cleared from circulation by immune-mediated mechanisms. Nearly 200 genes were differentially expressed in *P. yoelii* iRBCs on day 18 relative to day 10 of infection; the largest set in this study. On day 18, several genes encoding components of the cytoplasmic translation machinery were down-regulated while a second set associated with merozoite invasion were up-regulated. In *P. falciparum*, transcription of genes encoding the cytoplasmic translation machinery generally peaks in ring-stage parasites at ~12 hours post-invasion, followed by a marked down-regulation in trophozoite- and schizont-stage parasites. In contrast, transcription of genes involved in schizont rupture and merozoite invasion is relatively low through most of the asexual cycle, peaking at ~42 hours post-invasion [4,5]. Differences noted in gene expression in *P. yoelii* parasites on day 18 versus day 10 of infection may partially be explained by a modest shift noted in distribution of parasite stages in circulation toward increased schizonts and decreased trophozoites. It is also clear that infected blood isolated on day 18 contains damaged host cells and dying parasites, as well as free extracellular parasites. These factors likely contributed to a mixed alteration in expression profiles at this late time point during infection.

To focus on the role of the host immune response on parasite gene expression, two approaches to actively alter host immune pressure were taken. In the first approach, all antibody mediated immune pressure was removed by infecting B cell deficient J_H_D mice. It was expected that in the absence of antibodies, *P. yoelii* growth would be unchecked with an increase in the diversity of parasite populations and gene expression profiles. This did not occur as only seven genes were differentially expressed when *P. yoelii* parasites isolated from B cell deficient J_H_D mice were compared with those from immunocompetent control mice. In the second approach, immune pressure on *P. yoelii* 17X parasites was increased by immunizing mice with a preparation of parasite proteins associated with the membrane of infected reticulocytes. Here, immunization-induced antibodies were expected to select for the growth of parasites with altered gene expression patterns that included changes in members of the *yir* and *pyst-a* multigene families that encode proteins localized to the erythrocyte membrane. Unexpectedly, this did not occur as only 10 genes were differentially expressed. Even in these situations where the host immune response was experimentally manipulated, *P. yoelii* 17X gene expression proceeded unaltered. This is very reminiscent of the ‘hard-wired’ programme of gene expression observed with in vitro cultures of *P. falciparum*[4,5,46].

In addition to addressing global changes in gene expression, the expression of members of two multi-gene families in *P. yoelii*, the *yir* and *pyst-a* were of particular interest. Previous studies have shown that at a population level, many *yir* genes are transcribed during blood-stage infection, in a seemingly random order. Transcription at the level of individual parasites appears to be tightly controlled, with each parasite transcribing between one and three *yir* genes [55]. In the present study, significant differences in the expression of *yir* family members across experimental conditions were not observed, including the comparison of *P. yoelii* 17X day 10 and day 18 parasites. This is in agreement with the study by Cunningham *et al.* examining the expression of a subset of the *yir* repertoire on days 12 and 18 post-infection [20]. Of note, expression of a fairly large number of the *yir* genes (89–155) was consistently observed using signal intensity greater than the 50^th^ percentile on each array as a benchmark. These expressed *yir* genes were distributed across five previously reported phylogenetic groups, with no obvious bias toward any one group [56].

The expression of *pyst-a* genes followed a similar pattern to the *yir* family, with little to no differential expression between conditions. It appears that a large proportion of *pyst-a* family members are expressed in a mixed blood-stage infection. Whether *pyst-a* gene expression is more restricted in individual parasites has not been determined. No *yir* or *pyst-a* members were differentially expressed in wild-type versus J_H_D animals, in agreement with a study examining expression of a subset of *yir* members in wild-type versus Rag2 knockout mice, which lack mature B and T cells [20]. A more extensive analysis of *yir* and *pyst-a* expression during primary and secondary infections using a microarray approach would be of interest. It has been suggested that the polymorphic erythrocyte membrane antigens encoded by the *yir* and/or *pyst-a* multigene families may function as part of a parasite immune evasion strategy. For immune evasion, the current data suggest that changing the pattern of gene expression may be less important than showering the host immune system with a large repertoire of polymorphic antigens at any given time. In fact, prior immunization of mice with the *Py* RMP preparation may have further impeded the development of protective immune responses following challenge infection as these animals developed a persistent infection which was difficult to clear. In agreement with the current findings, members of the *cir* multi-gene family do not seem to be differentially expressed during the course of blood-stage *P. chabaudi* infection, but there is some indication that *cir* gene expression varies in parasites localized to different host tissues [21]. However, it is still possible that much like the *P. falciparum* EMP1 and the *var* gene family, the *yir* and/or *pyst-a* encoded proteins may possess specific functions required for parasite growth and development in vivo.

Overall, in vivo, *P. yoelii* 17X gene expression did not appear to be appreciably influenced by the host environment. These data in the *P. yoelii* 17X model differ from that reported by Daily *et al.*[7] who detected distinct patterns of gene expression in *P. falciparum* parasites isolated from malaria-infected patients. In light of these data, changes in the expression of genes encoding a set of mitochondrial proteins (n = 148), across all experimental conditions was examined. Significant changes that would be consistent with distinct physiological states or a response to environmental stress were not observed (Additional file 3). Although parasite gene expression was assessed in vivo, the *P. yoelii* model is still less complex than with *P. falciparum* infected human subjects. Here, a single cloned line of *P. yoelii* 17X that does not produce gametocytes was utilized to simultaneously infect genetically identical mice housed in environmentally controlled, specific pathogen free conditions. In some regards, this lack of gametocyte-stage parasites is advantageous, as detection of differential gene expression due to varying gametocyte levels across samples can be ruled out. On the other hand, the repeated passage of this *P. yoelii* 17X line in the vertebrate host and/or the lack of exposure to the mosquito vector could have altered regulatory mechanisms that control expression of certain gene subsets (i.e. *yir* or *pyst-a* genes). It is also possible that the greater diversity in human hosts (genetic, environmental) may have a greater influence on parasite gene expression than we observed in the *P. yoelii* model. Finally, each isolate of *P. falciparum* may exhibit unique elements of a ‘hard-wired’ programme of gene expression that can be detected when comparing isolates obtained from individual malaria patients. Co-infection of human subjects with such distinct *P. falciparum* clones will increase diversity in the overall *P. falciparum* gene expression profile detected in a single host and may allow preferential growth of *P. falciparum* clones in specific in vivo environments.

## Conclusions

The present study shows that gene expression patterns of blood-stage *P. yoelii* 17X parasites replicating in vivo are very stable and do not vary appreciably even at ascending and peak parasitaemia and in the presence or absence of host immune pressure. These findings support the notion of a ‘hard wired’ gene expression pattern in plasmodial species. Additionally, although a relatively large number of *yir* and *pyst-a* are expressed across the conditions studied here, there was no evidence of significant differential expression within these multi-gene families. Concurrent expression of a large number of polymorphic antigens in blood-stage malaria parasites may be a strategy to impede the development of protective immune responses.

## Abbreviations

CON-A/CM: Conserved, amino acid/carbohydrate metabolism; CON-AP/M: Conserved, apicoplast, mitochondrion; CON-CD: Conserved, cell division, DNA replication; CON-CTM: Conserved, cytoplasmic translation machinery; CON-ITV: Conserved, intracellular transport/vesicles; CON-PM: Conserved, protein folding, modification, degradation; CON-RS: Conserved, ribonucleotide synthesis; CON-SMI: Conserved, schizont, merozoite, invasion; CON-SP: Conserved, structural protein; CON-TM: Conserved, transcription machinery; CON-UF: Conserved, unknown function; RMP-MG: Rodent malaria parasites, multigene family; RMP-UF: Rodent malaria parasites, unknown function; PY-pyst-a: *P. yoelii* specific, *pyst-a* family; PY-yir: *P. yoelii* specific, *yir* family; PY-UF: *P. yoelii* specific, unknown function.

## Competing interests

The authors declare that they have no competing interests.

## Authors’ contributions

ACO conceived, designed and performed the experiments and wrote the paper. JMB conceived and designed experiments, analyzed the data, and wrote the paper. ABV analyzed the data. LWB analyzed the data and contributed reagents/materials. TMD contributed reagents/materials. All authors read and approved the final manuscript.

## Supplementary Material

Additional file 1 ***yir *and *pyst-a *oligonucleotides on *Plasmodium yoelii *DNA microarrays that mapped to a single gene.** In assessing the number of *yir* and *pyst-a* genes expressed in a given sample, only data obtained with *yir* and *pyst-a* oligos that mapped to a single gene were considered. Mapping data available on PlasmoDB [3] was used to focus the analysis on 464/859 *yir* oligos (top portion of Table) and 74/140 *pyst-a* oligos (bottom portion of Table) on *P. yoelii* DNA microarrays. Click here for file

Additional file 2 **Differential gene expression between *Plasmodium yoelii *17X parasites isolated from individual mice.** Three animals (M1, M2 and M3) were infected with *P. yoelii* 17X iRBCs from a single donor mouse and gene expression analysed using *P. yoelii* DNA microarrays. Of three possible pair-wise comparisons, significant differential gene expression was only seen between M2 and M3 (black text) and M1 versus M3 (blue text). For each differentially expressed gene, oligo ID and gene name are listed [3]. Log_2_ Fold Changes and adjusted *p* -values are also included, as are the predicted number of amino acids (AA) and molecular weight in Daltons (MW). Where available, *P. falciparum* orthologs and associated Plasmodb.org accession numbers are included (Pf Ortholog # and Pf Name). Functional groupings (Category) are listed at the far right of the Table. For detailed information regarding data analysis and gene categorization, please see the Methods. Click here for file

Additional file 3 **Comparative expression of *Plasmodium yoelii *genes encoding mitochondria associated proteins.** Data on the differential expression of genes encoding mitochondria associated proteins is shown for all comparisons. The gene set included *P. yoelii* orthologs of putative *P. falciparum* mitochondrial proteins [7,41] Mather and Vaidya, unpublished data] for which signal intensity across arrays was consistently above the 50^th^ percentile. Shown are the *P. falciparum* Gene ID and Name, *P. yoelii* Oligo ID and Name and for each comparison, the corresponding the log_2_ fold change and adjusted *p* -value. Log_2_ fold change values >1 are shaded in red, log_2_ fold change values < −1 are shaded in green and adjusted *p* -values <0.01 are shaded in yellow. Click here for file

Additional file 4 **Differential gene expression between parasites isolated from a single donor mouse and three individual animals.** Three animals (M1, M2 and M3) were infected with *P. yoelii* 17X iRBCs from a single donor mouse (D) and gene expression analysed using *P. yoelii* DNA microarrays. Three pair-wise comparisons were possible: D versus M1 (black text), D versus M2 (blue text) and D versus M3 (red text). For differentially expressed genes, oligo ID, gene name, log_2_ fold change, adjusted *p* -value, predicted number of amino acids, predicted MW, available *P. falciparum* ortholog information and functional categories are provided as described for Additional file 2. Click here for file

Additional file 5 **Differential gene expression between *Plasmodium yoelii *17X parasites isolated from independent infections.** Groups of animals were infected on four separate occasions (I1, I2, I3 and I4) using four separate donor animals and gene expression analysed using *P. yoelii* DNA microarrays. Six possible pair-wise comparisons were considered: I1 versus I2 (black text), I1 versus I3 (blue text), I1 versus I4 (red text), I2 versus I3 (green text), I2 versus I4 (purple text) and I3 versus I4 (blue text). For differentially expressed genes, oligo ID, gene name, log_2_ fold change, adjusted *p* -value, predicted number of amino acids, predicted MW, available *P. falciparum* ortholog information and functional categories are provided as described for Additional file 2. Click here for file

Additional file 6 **Differential gene expression on days 10/14, 10/18 and in the absence/presence of host antibody responses.** Groups of wild-type animals were infected with *P. yoelii* 17X iRBCs and parasite RNA was isolated on day 10 (D10), day 14 (D14) or day 18 (D18) post-infection. Immunologically intact (WT) and B-cell deficient J_H_D (J_H_D) animals were similarly infected and parasite RNA was isolated on day 10. A third set of animals was immunized three times with a preparation of *P. yoelii* reticulocyte membrane proteins plus Quil A as adjuvant (RMP) or with Quil A alone (QA) prior to *P. yoelii* 17X challenge. Parasite RNA was then isolated on day 10 (QA) or day 12 (RMP) post-infection. Gene expression was analysed using *P. yoelii* DNA microarrays and the following comparisons were made: D10 versus D14 (black text), D10 versus D18 (blue text), WT versus J_H_D (red text), and QA versus RMP (green text). For differentially expressed genes, oligo ID, name, log_2_ fold change, adjusted *p* -value, predicted number of amino acids, predicted MW, available *P. falciparum* ortholog information and functional categories are provided as described for Additional file 2. Click here for file
